# The Bioaccessibility of Antioxidants in Black Currant Puree after High Hydrostatic Pressure Treatment

**DOI:** 10.3390/molecules25153544

**Published:** 2020-08-03

**Authors:** Urszula Trych, Magdalena Buniowska, Sylwia Skąpska, Szymon Starzonek, Krystian Marszałek

**Affiliations:** 1 Department of Fruit and Vegetable Product Technology, Institute of Agricultural and Food Biotechnology, 02532 Warsaw, Poland; sylwia.skapska@ibprs.pl; 2 Department of Dairy Technology, Institute of Food Technology and Nutrition, University of Rzeszow, Ćwiklinskiej 2D St., 35601 Rzeszów, Poland; mbuniowska@ur.edu.pl; 3 Institute of High Pressure Physics, Polish Academy of Sciences, 29/37 Sokołowska St., 01142 Warsaw, Poland; starzoneks@unipress.waw.pl; 4 Department of Food Technology and Human Nutrition, Institute of Food Technology and Nutrition, University of Rzeszow, Poland, Zelwerowicza 2D, Rzeszow 35-601, Poland

**Keywords:** bioaccessibility, high hydrostatic pressure, black currant, vitamin C, anthocyanins, antioxidant activity, in vitro digestion model

## Abstract

The aim of the study was to investigate the effect of high-pressure processing (HPP) and thermal processing (TP) on the bioaccessibility of vitamin C and anthocyanins as well as changes in the antioxidant capacity (AC) using ABTS+• and DPPH• tests on blackcurrant (*Ribes nigrum* L.) puree during the steps in the digestive process. The puree was subjected to HPP at 200, 400, and 600 MPa for 5 min (room temperature) or TP at 85 °C for 10 min. The controls were untreated puree (P) and fruit crushed in a mortar (M). All the samples were digested in a static in vitro digestion model, including the mouth, stomach, and small intestine, and subjected to dialysis. The vitamin C, anthocyanin, and antioxidant capacity were monitored at each step of the digestion process. The potential bioaccessibility of the antioxidants studied was calculated in relation to the undigested sample. TP and HPP enabled a high content of vitamin C, anthocyanins, and AC to be maintained. After simulated digestion in the small intestine, a significant decrease was observed in the vitamin C and anthocyanins (approximately 98%) content. However, a high stability (approximately 70%) of both compounds was noted at the gastric stage. HPP and TP significantly affected the potential bioaccessibility of vitamin C and anthocyanins, although the bioaccessibility of both compounds in the samples treated using HPP was higher than when using TP. Moreover, the potential bioaccessibility of vitamin C after HPP treatment (400 and 600 MPa) was higher than the bioaccessibility calculated for the M and P control samples. TP and HPP treatment negatively affected anthocyanin bioaccessibility after dialysis. The most favorable pressure was 400 MPa, as it allowed maintaining the best antioxidant activity after digestion.

## 1. Introduction

Berries, including blackcurrants (*Ribes nigrum L.*), are distinguished by their excellent nutritional values. Due to their mineral, vitamin, and antioxidant content, they are recommended from a nutritional point of view. They are a rich source of vitamin C and anthocyanins responsible for their intensely dark red color [1]. Blackcurrants contain 160–285 mg/100 g of ascorbic acid; therefore, consuming approximately 20 g of this fruit provides the recommended daily allowance (RDA) for vitamin C [2]. Depending on the variety, region, ripeness, growing, and storage conditions, the concentration of polyphenols can be as much as from 500 to 1342 mg/100 g, and most of them are anthocyanins (160–411 mg/100 g) [3,4]. Blackcurrants are very good material for the production of juices, frozen foods, jams, and tinctures and can also be eaten as fresh fruit. Their many nutritional compounds also make them an interesting ingredient for the functional foods sector [2]. Their high content of antioxidants could help in the prevention of tumors and cardiovascular diseases. Anthocyanins are also responsible for anti-inflammatory, antimicrobial, and neuroprotective effects, the induction of cell apoptosis, and may help in maintaining eye health [5,6]. The free radical scavenging mechanisms, and inhibiting the formation of carcinogenic nitrosamine in the stomach, contribute to the antimutagenic and antioxidant effect of vitamin C [7]. Epidemiological studies in rats have proven that the long-term low-dosage administration of vitamin C prior to middle cerebral artery occlusion had a neuroprotective effect and may protect the brain against damage and strokes [8].

The bioavailability of nutrient and bioactive compounds is defined as the part of a substance administered for digestion and it is capable of being absorbed and available for use in physiological functions [9]. The concept of “bioavailability” includes availability for absorption, which is determined as “bioaccessibility”, absorption, tissue distribution, and bioactivity. It is not essential for all antioxidants to be fully absorbed from the digestive tract into the systemic circulation in order to fulfill their function and bring beneficial effects for the human body. By remaining in the intestines and colon, they can protect epithelial cells from oxidative DNA damage and cancerous changes associated with the release of free radicals [10].

Due to the difficulties associated with performing and comparing in vivo tests, static in vitro models are often used to assess bioaccessibility. In vitro tests map optimal conditions in the gastrointestinal tract, help to avoid ethical problems, and enable us to understand certain mechanisms that guide the digestive processes and the absorption of bioactive components and their potential bioaccessibility [11,12].

Numerous studies have shown the positive effects of high-pressure processing (HPP) on the stability of bioactive ingredients, compared to thermal processing, and the increase in the bioaccessibility of selected nutritional compounds was confirmed. HPP may affect plant tissue structures and increase the extractability of bioactive ingredients, simultaneously having a positive effect on their initial stability. Moreover, this treatment can disrupt the structure of the ingredients, both bioactive (polyphenols, folates) and those capable of binding them (such as pectin), thus making them more available. Therefore, high hydrostatic pressure may be a useful method of preservation in designing functional foods with high nutritional values [13,14]. According to our best knowledge, there are a limited number of studies on the influence of HPP on the bioaccessibility of anthocyanins and vitamin C, as well as changes to the antioxidant capacity (AC), during the digestive steps; therefore, the aim of this study was to evaluate the influence of HPP conditions on the potential bioaccessibility of anthocyanins, vitamin C, as well as changes in the AC after simulated digestion in the mouth, stomach, and small intestine. Moreover, the passive transport of metabolites through the artificial membrane was investigated after dialysis. The differences between the HPP and thermal processing (TP) techniques, as well as between the various degrees of tissue disruption (puree and crushed fruits), were also highlighted.

## 2. Results and Discussion

### 2.1. Effect of Processing on the Bioaccessibility of Vitamin C

The vitamin C content in blackcurrants before digestion was the highest in fresh fruit crushed in a mortar (M), followed by untreated (P) and HPP treated puree at 200 MPa (HPP200) (Table 1). All the processes were insignificant regarding the degradation of vitamin C (except for the HPP600 sample) before digestion, which indicates that the vitamin C in blackcurrants is quite resistant to thermal treatment. This may be due to the fruit’s natural high acidity. In the control samples, vitamin C occurred only in the form of L-ascorbic acid, and only this bioactive form of the vitamin was analyzed.

The concentration of L-ascorbic acid decreased by approximately 50% in all the samples after salivary digestion. The significant degradation in vitamin C could be connected with the raising of the pH value to 6.75 ± 0.20. Furthermore, it was evident that vitamin C was less stable in the samples treated with HPP at 200 MPa as well as TP. This phenomenon may be connected with the oxidation of L-dehydroascorbic acid to L-dehydroascorbic acid at a higher pH in the mouth. The most stable form of vitamin C was in the M samples, which was probably due to the larger fruit particles that can protect less available nutrients inside the tissue. During the digestive phase in stomach conditions, the vitamin C concentration increased significantly compared to the salivary phase. The highest concentration of vitamin C was observed in the M and HPP600 samples. Higher pressures promoted the reversibility of the degradation reactions, and lower concentrations of vitamin C were found when lower pressures were applied. In the unprocessed P samples, the vitamin C concentration was lower than in the samples treated using HPP at 400 and 600 MPa, and higher than in the same samples treated using 200 MPa and treated with TP. The acidic environment of the stomach is able to promote the stability of vitamin C, which is also confirmed by other studies—however, in the context of polyphenols [15].

**Figure 1:**
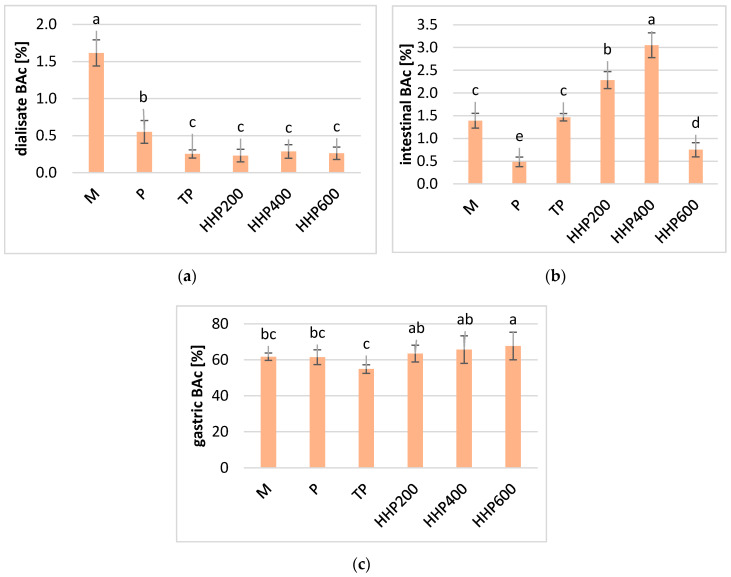
Relative bioaccessibility of total vitamin C in blackcurrants after different processing: M–crushed in a mortar; P–untreated puree; TP–thermal pasteurized puree; HPP200–high pressured puree at 200 MPa; HPP400–high pressured puree at 400 MPa; HPP600–high pressured puree at 600 MPa; (**a**) in the dialysate stage; (**b**) in the intestinal stage; (**c**) in the gastric stage; small letters a–e indicate statically significant differences in the mean values between samples at a confidence level of α = 0.05.

Vitamin C was largely destroyed as a result of digestion in the small intestine (approximately 98–99%), irrespective of the type of processing and the conditions; whereas in dialysate, vitamin C was only detected in the samples treated using HPP at 400 and 600 MPa. The highest content of the vitamin was found in HPP600 samples (up to 4.18 mg/100 g), and the lowest content was found in the TP sample (2.35 mg/100 g). Such a large degradation of vitamin C may by caused by the high pH in the small intestine. Vitamin C is sensitive to environmental changes and is unstable in alkaline and neutral pH, at high temperatures, and in the presence of oxygen and some metal ions [16,17]. Other authors have also confirmed that vitamin C degraded under intestinal conditions, showing a decrease of 91% after the digestion of fresh broccoli [7]. Other studies reported a significant degradation in vitamin C (>95%) in pomegranate juice, after in vitro digestion at the small intestine stage [18]. Studies involving vitamin C and phenolic compounds have also confirmed their high stability in stomach conditions (a recovery of more than 75%) as well as a decline in their concentration during intestinal digestion [19]. According to our best knowledge, there is currently no research describing the changes in vitamin C at all the stages of digestion, including salivary digestion. The relative bioaccessibility (BAc) calculated for vitamin C after dialysis in relation to the non-digested sample is slight (Figure 1a). The highest BAc at this stage was calculated for vitamin C in samples treated using HPP at 400 and 600 MPa (about 1%). The BAc of vitamin C from the small intestine fraction was slightly higher, ranging from 1% in sample T to 2% in TP and HPP600-treated samples (Figure 1b). The BAc of vitamin C in samples treated under pressure at 400 and 600 MPa was higher than the BAc calculated for unprocessed M and P puree. This means that HPP may increase the BAc of vitamin C, which is probably due to the enhanced extraction of this compound from the tissue. HPP treatment may increase the extractivity of bioactive ingredients; however, the type of food matrix is of great importance to the further effect of increasing BAc [14,20]. It transpired that a pressure of 200 MP was insufficient to increase the BAc of vitamin C. This phenomenon may be justified by the low degradation of tissue enzymes such as polyphenol oxidases and peroxidases, which may accelerate the degradation of vitamin C. On the other hand, vitamin C is metabolized at a high pH and may also be absorbed by the epithelial lining of the small intestinal as metabolites. Ascorbic acid can be absorbed via a sodium-dependent active transporter (SVCT1) mainly in the ileum and jejunum, while the absorption of dehydroascorbic acid is facilitated by diffusion in the duodenum and jejunum using glucose transporters. Generally, it is clear that compounds undergoing dialysis would be available for absorption in the human body, but in the case of vitamin C, it is highly probable that ascorbic acid is absorbed into the human cardiovascular system from the stomach or just at the beginning of the small intestine [21]. The BAc of vitamin C after digestion in the stomach phase was very good and was within the limits—from 60% for TP and HPP200-treated samples, and up to 90% for M and HPP600 samples (Figure 1c). It should be emphasized that vitamin C from HPP-treated samples at 600 MPa had a greater BAc than the P sample, which indicated that the HPP technique may increase the BAc of vitamin C. However, TP significantly decreases the Bac to below the BAc recorded for the P and M samples.

There is limited research on the effect of HPP on the bioaccessibility of vitamin C. Aschoff et al. (2015) assessed the BAc of vitamin C in orange juices and observed that thermal pasteurization increased the BAc of vitamin C compared to fresh orange juice [22]. Cilla et al. (2012) verified the effect of short thermal treatment (90 °C, 30 s) on vitamin C BAc in fruit juices, with the addition of various types of milks compared to HPP treatment at 400 MPa and 40 °C for 5 min. They concluded that HPP, in contrast to thermal treatment, enabled the retention of vitamin C at a level similar to fresh juices. However, the retention of vitamin C did not contribute to an increase in the BAc of this component. The authors concluded that HPP can promote the formation of aggregates of whey protein in juices with the addition of milk and protect against the degradation of vitamin C by increasing viscosity, which may influence the reduction in the BAc of ascorbic acid molecules in the in vitro digestion model [23]. In another study, an increase in the BAc of vitamin C in fruit beverages with water, milk, or soymilk treated using HPP (400 MPa, 5 min, 40 °C) compared to thermally treated (90 °C, 60 s) samples was reported. The authors confirmed that HPP may contribute to the inactivation of enzymes responsible for vitamin C oxidation such as ascorbate oxidase and peroxidases, whereas short-term heating may not be sufficient to inactive these enzymes [24].

### 2.2. Effect of Processing on the Bioaccessibility of Anthocyanins

According to the data presented in Table 1, the content of the sum of anthocyanins in blackcurrants before digestion was the highest in crushed fruits (sample M) and was 736.70 mg/kg of raw material. The sum of anthocyanins in the other samples was between 638.04 and 601.98 mg/kg in P and TP treated puree, respectively. The total anthocyanin content was higher in HPP-treated samples than in the TP, M, and P-treated ones, thus confirming that anthocyanins in blackcurrants are quite stable at high pressures [25]. Four main anthocyanin monomers were found: delphinidin-3-o-rutinoside (Df 3-o-rut), cyanidin-3-o-rutinoside (Cy 3-o-rut), delphinidin-3-o-glucoside (Df 3-o-glu), and cyanidin-3-o-glucoside (Cy 3-o-glu), and the same anthocyanin composition was found by other authors [26]. Cy 3-o-rut, which is the second most common anthocyanin in the cultivar of blackcurrant studied, is also known to be the most stable after thermal treatment [27].

The salivary digestion of all the samples caused a significant degradation in the anthocyanins (Table 1). Similarly, according to Carbonell-Capella et al., 2015. In beverages based on exotic fruits, the recovery of total anthocyanins detected after the salivary phase was significantly lower than those in the non-digested samples [28]. It is well known that anthocyanins are very sensitive to pH changes, and this reaction is reversible [25]. The largest decrease in these pigments was noted in the TP sample, reaching a value of 178.15 mg/kg, whereas the lowest was in the HPP200, M, and T samples: 220.23 mg/kg, 257.43 mg/kg, and 251.94 mg/kg, respectively. The most stable anthocyanins were in the HPP400 and HPP600 samples and amounted to 287.85 mg/kg and 277.29 mg/kg. As a result of digestion in an acidic stomach environment, the anthocyanin content increased significantly, but the concentration of this pigments reached 55–65% of the concentrations noted in the samples before the first step of digestion. A similar relationship was also found in another study. After the simulated digestion of grape samples in the mouth phase, the anthocyanin content was lower than in the control sample, before digestion, whereas it increased significantly in the gastric phase. Ultimately, 45% of the anthocyanins were available after the gastric stage. The authors concluded that this phenomenon is related to the type of food matrix, since there was no similar relationship in wine samples [15].

**Figure 2:**
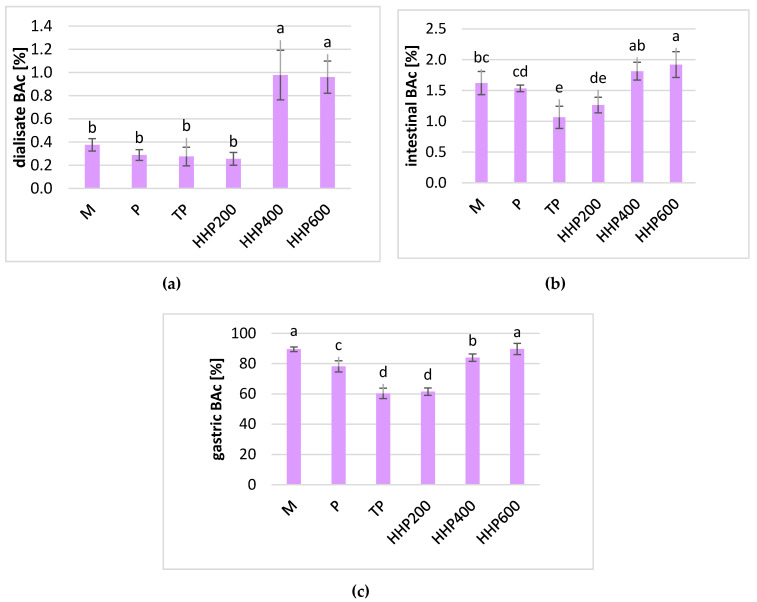
Relative bioaccessibility of total anthocyanins in blackcurrants after different processing: M–crushed in a mortar; P–untreated puree; TP–thermal-treated puree; HPP200–high-pressured puree at 200 MPa; HPP400–high-pressured puree at 400 MPa; HPP600–high-pressured puree at 600 MPa; (**a**) in the dialysate stage; (**b**) in the intestinal stage; (**c**) in the gastric stage; small letters a–d indicate statically significant differences in the mean values between samples at a confidence level of α = 0.05.

Simulated intestinal digestion resulted in a drastic decrease in anthocyanin contencentration. The highest degradation was noted in the P, HPP600, and TP sampled and reached from 0.5% to 1.5% compared to undigested samples. The best anthocyanin retention was noted in the M, HPP200, and HPP400 samples, where the concentration of these pigments was as high as 3% of the original value. A similar concentration of anthocyanins was detected in the dialysate obtained from untreated M and P samples. All the treatment processes resulted in a significantly lower absorption of anthocyanins after dialysis by artificial membranes. Similarly to vitamin C, the Bac of anthocyanins calculated according to the ratio of the substance in the dialysate and non-digested samples was very low (Figure 2a). For all the treated samples, the BAc was approximately 0.2–0.3%, and no statistically significant differences were observed. For samples M and P, the Bac of anthocyanins was higher and reached 1.62% and 0.55%, respectively. Taking into consideration the content of the substance digested at the gastric stage, the BAc of anthocyanins was statistically significantly lower in the TP samples (55%) compared to the HPP-treated samples (Figure 2c). The BAc of anthocyanins in the M and P samples was significantly lower compared to the HPP-treated samples, but it was still higher than the BAc calculated for the TP samples.

Previous studies confirm the high stability of anthocyanins in the gastric digestion phase and a large decrease in BAc after intestinal digestion [15,29,30,31,32]. The authors justified this phenomenon by the break in the anthocyanin ring when the pH of the environment changes from acidic (pH 2) to alkaline (pH 8). Only delphinidin- and malvidin-6-acetoyl 3-glucosides were relatively stable to changes in the pH and showed far less degradation [30]. In the bioaccessibility studies of polyphenols (including anthocyanins) in apples, other authors observed a significant degradation in anthocyanins after digestion at the intestinal stage. This was explained by the pH changes from acidic in the stomach to alkaline in the small intestine, as well as by the action of pancreatin and bile. The release of anthocyanins from the fruit matrix is most effective during gastric digestion [15,29]. Bouayed et al. (2011) noted that most polyphenols and flavonoids were already available in the gastric phase, but not all polyphenols detected in the intestinal stage were available for absorption under the conditions simulated by dialysis experiments. Studies of anthocyanin derived from blueberries proved that the delphinidine derivatives were unstable under gastrointestinal digestion. They were not detected after digestion and showed the lowest BAc. The differences in the availability of various anthocyanins may be related to the number of –OCH3 groups present in the different molecules. It has been noted that a larger number of these groups promote bioavailability, since larger structures may contribute to less degradation and susceptibility to conversion to nondetectable (colorless) forms [33].

Anthocyanins are very susceptible to degradation under the influence of temperature, light, pH changes, the presence of oxygen, sugars, sulfites, ascorbic acid, metal ions, and other pigments. They are also sensitive to the activity of tissue enzymes (β-glucosidase, polyphenoloxidase, and peroxidase), and microorganisms [25]. At a pH below 3, anthocyanins are found in the form of red flavylium cations. As the pH increases to 4, these cations are transformed into purple-violet quinoidal bases. At pH 4–6, colorless hemiketal forms may occur and also yellow retro-chalcones, resulting from ring opening. Furthermore, anthocyanins may undergo co-pigmentation reactions that occur in the presence of some compounds, such as other phenols and metal ions (including magnesium, copper, and aluminum), which increases their stability. Anthocyanins may also be easily transformed into insoluble polymeric brown pigments, which are liable to degradation at elevated temperatures, causing the polymerization of monomeric anthocyanins to form brown compounds [25]. Anthocyanin bioaccessibility from foods is very limited. Even after consuming large amounts of these compounds, their maximum plasma concentration is at a level of several tens of nM and occurs just over an hour after ingestion. Moreover, the concentration of anthocyanins in the urine is less than 0.1% of the initial dose. However, it should be noted that studies often do not take into account all anthocyanin derivatives that may have arisen in the metabolic pathway, and moreover, they often forget about the possibility of biliary excretion back into the gastrointestinal tract [34,35]. The low bioaccessibility of anthocyanins may also be connected with their bonding with other plant tissue components, such as cellulose, pectin, and fiber [35]. As the blackcurrant is known to be a fruit containing a large amount of pectin (2.5 g/100 g), this hypothesis is highly probable [36].

However, anthocyanins can also be absorbed in a glycoside form through the gastric mucosa, as has been demonstrated in several studies [37,38]. Studies conducted on rats fed a diet enriched with blackberry anthocyanins have shown that anthocyanins and their metabolites can accumulate in the tissues of organs such as the bladder, prostate, testicles, heart, and adipose tissue. This demonstrates their bioavailability from the gastrointestinal tract and the circulatory system to targeted organs. Anthocyanins can be absorbed from the stomach and intestines, but they can also reach the colon, where they are metabolized by the microbiota, which confirmed the hypothesis that these compounds do not have to be stable in the small intestine [39].

The mechanisms describing the metabolism and absorption of anthocyanins are not yet fully understood. There are indications that they may even be absorbed in the mouth, as anthocyanins can be present in the blood plasma 5 min after their contact with the oral epithelial tissue. In addition, there are similar transporters in the mouth as in the intestine that can contribute to the absorption of anthocyanins from the oral cavity [40]. The gastrointestinal microflora plays also an important role in the digestion and absorption of anthocyanins [41]. Research results indicate that colon fermentation reduces the antioxidant activity of polyphenols and their potential to inhibit cancer cells [30]. In spite of the generally low bioavailability of anthocyanins, epidemiological studies indicate their positive effect on the body. This may be due to the biological activity of the metabolites and catabolites produced by microorganisms, as well as their potential synergistic interaction with other compounds [39].

It has been shown that food processing, including high-pressure techniques, can increase the bioaccessibility and bioavailability of antioxidants through the chemical or physical modification of food. The application of HPP helps in the retention of thermolabile antioxidants during processing and therefore favors more effective absorption in the gastrointestinal tract [35]. To the best of our knowledge, there is currently a lack of research on the effects of high pressure on the bioaccessibility of anthocyanins. Rodríguez-Roque et al. (2015) demonstrated that HPP treatment (400 MPa, 5 min, 40 °C) improved the bioaccessibility of total phenols compared to thermal treatment and control samples. The authors explained that HPP may affect the bioaccessibility of phenolic compounds by intervening with their structure (e.g., hydroxylation, methylation, isoprenylation, dimerization, and glycosylation). Phenolic derivatives may also be formed through the partial degradation of linked forms. HPP can also improve the bioaccessibility of active ingredients by affecting the structure of fiber, fats, and phytosterols, which are often associated with other food components, disturbing their absorption [24].

### 2.3. Effect of Processing on the Antioxidant Capacity of Blackcurrant Puree in a Simulated Digestive System

Measuring the antioxidant capacity (AC) of food products with 2,2′-azino-bis(3-ethylbenzothiazoline-6-sulfonic) acid diammonium salt (ABTS^+•^) and 2,2-diphenyl-1-picrylhydrazyl (DPPH^•^) radicals relies on a single electron transfer mechanism [42]. In the control samples, the AC measured with ABTS^+•^ was between 27.03 and 31.82 µM/mL Trolox Equivalent Antioxidant Capacity (TEAC) in the TP and HPP600 samples, respectively. A similar trend was observed using DPPH^•^ radicals, but the values were from 45.11 to 45.89 µM/mL TEAC, respectively (Table 1).

The highest AC in the salivary digestive fraction measured with ABTS^+•^ was noted for HPP-treated samples at 400 and 600 MPa, whereas the type of processing and conditions were insignificant in tests using DPPH^•^. The lowest AC was noted in samples treated using TP and HPP at 200 MPa. The AC measured with the ABTS^+•^ test increased significantly in all the samples compared to the undigested samples, which indicates that digestion in the salivary step may increase the antioxidant potential of the nutritional compounds found in blackcurrants. By contrast, the method using the DPPH^•^ radical noted a 50% decrease in AC in all the samples, and these values did not show statistically significant differences.

Digestion in the stomach caused a further increase in AC measured with ABTS^+•^ in all the samples. The greatest changes were noted for the TP and HPP200 samples, where AC increased by almost 26% and 13%, respectively, compared to the salivary digestion stage. Despite the significant growth, their value was still lower than in the other samples. The highest value of AC was recorded in the HPP400 sample, reaching 42.02 µM/mL TEAC. Similarly, using the DPPH^•^ method, an increase in AC was observed after gastric digestion. The highest increase occurred in the HPP400 and HPP600 samples, 28% and 23%, respectively.

During simulated digestion in the small intestine, there was a significant decrease in AC in all the samples. It was probably associated with a decrease in the vitamin C and anthocyanin content. Under the ABTS^+•^ method, the decrease ranged from 66% in M to 92% in the TP samples. The M sample showed the highest AC, followed by HPP400 and P, whereas the pasteurized samples had the lowest AC. There were similar relationships in the dialysate fraction. In most of them, an increase in AA was observed, indicating that the bioaccessible fraction contains metabolites of the tested active ingredients, which was characterized by high AC. In addition, a pressure of 400 MPa also had the greatest beneficial effect on the AA of the fruit product.

Rodríguez-Roque et al. found that the use of HPP reduced hydrophilic antioxidant activity (measured by the DPPH^•^ method) in fruit and soy-fruit beverages, but not below the AC noted for heat-treated samples. Depending on the matrix used, HPP caused an increase (in juices with milk), did not change (in juices with soymilk), or caused a decrease (in juices with water) in AC after digestion compared to the control samples, which indicates that the AC of food components is highly dependent on the food matrix. However, HPP always provided a higher AC than heat treatment in this experiment. The authors concluded that processing can cause changes in the structure of the analyzed substances, reducing or increasing their activity. The oxidation of certain bioactive compounds present in food may be the reason for the decrease in the AC of the food matrix after processing. However, processing can improve the concentration of antioxidants by their release from the food matrix or due to the inactivation of degradative enzymes [24]. In the research conducted on apples and algarrobo seeds, Briones-Labarca et al. also noticed an increase in the total antioxidant capacity (DPPH^•^ method) after applying HPP treatment (500 MPa, 10 min). Processing contributed to the release of antioxidants and an increase in their solubility after breaking the cell wall structure [43,44]. Anthocyanin bioaccessibility and antioxidant activity (ORAC method) in tart cherry extracts with the addition of mineral clay were also studied. Despite a large decrease in the anthocyanin content after digestion under duodenal conditions, an almost twofold increase in antioxidant activity (ORAC) was noted. This may indicate that the metabolites formed after the breakdown or transformation of anthocyanins may still have antioxidant activity and that this activity is much stronger than before digestion [45].

## 3. Materials and Methods

### 3.1. Reagents and Solvents

The following reagents, enzymes, and laboratory equipment were purchased at Sigma-Aldrich (St. Louis, MO, USA): mucin from the porcine stomach—type II, α-amylase, heat-stable, (TDF-100A, 24975 U/mL), pepsin from porcine gastric mucosa (250 U/mg solid), pancreatin from the porcine pancreas (8 × USP specifications), bile extract from porcine, sodium dodecyl sulfate—ACS reagent, sodium bicarbonate ≥99.5% used to simulate digestion, dialysis tubing cellulose membrane (average flat width 25 mm), 2,2′-azino-bis(3-ethylbenzothiazoline-6-sulfonic) acid diammonium salt (ABTS^+•^ radical), 2,2-diphenyl-1-picrylhydrazyl (DPPH^•^ radical), (±)-6-hydroxy-2,5,7,8-tetramethyl-chromane-2-carboxylic acid (Trolox), DL-dithiothreitol (HPLC) (DTT), phosphoric acid 85%, acetonitrile (HPLC), formic acid ≥95.0% and sodium hydroxide pellets ≥98.0%, (NaOH).

Other reagents were obtained from Chempur (Piekary Śląskie, Poland), such as di-sodium hydrogen phosphate anhydrous pure p.a. ≥99.0% (Na_2_HPO_4_), di-potassium hydrogen phosphate (K_2_HPO_4_), sodium chloride pure p.a. ≥99.9% (NaCl) and di-sodium edetate standard solution 0.01 mol/L (EDTA). Hydrochloric acid pure p.a. ACS reagent 37% (HCl) and potassium peroxodisulfate ≥99.0% were purchased from Honeywell Fluka (Seelze, Germany). Ethanol (96% CZDA) and methanol (HPLC grade) came from Avantor (Gliwice, Poland).

### 3.2. Raw Materials

#### 3.2.1. Blackcurrant Fruit Processing

The research material were frozen blackcurrants (*Ribes nigrum L.*) of the Tisel cultivar bought from a local market. Whole raw materials were divided into two parts. One part of the fruit was subjected to manual crushing (Ø > 5.0 mm) with a mortar (M). The other part was thawed and processed into puree (P) (Ø < 0.2 mm) with a colloid mill (Rietz, San Francisco, CA, USA). The puree obtained was divided into two subsequent parts and packed into 100 mL glass jars or 50 mL high-density polyethylene (HDPE) bottles. The glass jars were pasteurized in a laboratory bath pasteurizer (Labo Play, Bytom, Poland) at 85 °C for 10 min (TP). The puree packed in HDPE bottles was subjected to high-pressure processing (HPP) in a high-pressure U4000chamber (UNIPRESS, Warsaw, Poland) at 200, 400, and 600 MPa (HPP200, HPP400, HPP600) and room temperature for 5 min. To limit adiabatic heating, all the bottles were cooled in an ice bath immediately after HPP. All the samples were frozen at −18 °C before analysis.

#### 3.2.2. Extraction of Bioactive Compounds from Raw Materials

An extraction of bioactive compounds was prepared from all the samples before and after processing as well as after digestion in salivary and gastric steps. The intestinal fraction and samples after dialysis were analyzed directly without additional extraction. About 0.5 g of the sample was extracted by shaking for 5 min at 400 RMP (DLAB, Labindex, SK-0330-Pro, Warsaw, Poland) with 80% methanol solution acidified with 0.1% HCl and by ultrasonication for 5 min (ultrasonic bath, MKD-6, Warsaw, Poland). The supernatant was centrifuged for 5 min and 10,342× *g* (centrifuge, Rotina 380R, Hettich, Tuttlingen, Germany) at 4 °C. Extraction was repeated six times to a final supernatant volume of 50 mL.

### 3.3. In Vitro Digestion, Dialysis, and Calculation of Bioaccessibility

In vitro gastrointestinal digestion was conducted according to method presented by Minekus et al., 2014 [46] and Buniowska et al. (2017) [47] with slight modifications. All samples were digested in triplicate, and distilled water was used instead of blackcurrants for the blank sample. Simulated digestion was started from the salivary step. Fifty grams of each sample was transferred to a dark glass bottle (100 mL) and mixed with 5 mL of a saliva enzyme solution (2.38 g Na_2_HPO_4_, 0.19 g K_2_HPO_4_, 8 g NaCl, 100 mg mucin, and α-amylase of 200 U/L enzyme activity, dissolved in 1 L of distilled water). The pH was adjusted (pH meter HI 211, Hanna Instruments, Woonsocket, RI, USA) to 6.75 ± 0.20 by the addition of HCl (12 mol/L) or NaOH (2 mol/L), and the solution was incubated in a shaking water bath (Labo Play, SWB 8N) at 37 °C and 90 RPM for 10 min. To start the simulation of the gastric phase, 13.08 mg pepsin was added, and the pH was adjusted to 2.0 by the addition of HCl (12 mol/L). The mixtures were incubated in a shaking water bath (37 °C/90 RPM) for 2 h in the dark. Twenty grams of the sample from the stomach stage was transferred to a fresh dark glass bottle and titrated with NaOH (2 mol/L) to pH 5.00 ± 0.20 to initialize the intestinal phase. Then, 5 mL of pancreatin (1 g/L) and bile (25 g/L) dissolved in distilled water were added. Finally, the dialysis membrane filled with 25 mL NaHCO_3_ solution (0.5 M, pH 7.5) was immersed in the mixture for the dialysis step. Dialysis cellulose membranes were prepared the day before by boiling in 0.01 mol/L EDTA (with 2% NaHCO_3_ and 0.1% sodium dodecyl sulfate) for 10 min. The mixtures obtained were incubated again for 2 h in the shaking water bath under the previous conditions and cooled in the ice bath for 10 min. Samples were collected from each step of the simulated digestion for analysis. Bioaccessibility (BAc) was calculated according to Equation (1).

BAc [%] = 100 × (BC_digested_/BC_non-digested_) (1)

where BAc—bioaccessibility of bioactive compound; BC_digested_—the concentration of bioactive compound in the digested sample; BC_non-digested_—the concentration of bioactive compound in the non-digested sample.

### 3.4. Chemical Analysis

#### 3.4.1. Determination of L-Ascorbic Acid (AA)

Determination of the vitamin C content was performed according to the method presented by Odriozola-Serrano et al. (2007) [48]. The sample was transferred into a vial after appropriate dilution by 0.01% m-phosphoric acid and filtering on the disposable syringe filter (0.45 µm, Macherey-Nagel, Düren, Germany). A Waters chromatographic system (Milford, MA, USA) equipped with a 2695 Separations Module and 2995 Photodiode Array Detector was used. A column—Sunfire C 18, 5 µm, 4.6 mm × 250 mm with reversed phase and Sunfire C18 Sentry guard insert, 5 µm, 4.6 mm × 20 mm (both Waters)—was thermostated at 25 °C. Samples were eluted isocratically with 0.01% m-phosphoric acid as an eluent at a flow rate of 1 mL/min for 10 min. The AA was quantified using UV absorption at 245 nm. 

#### 3.4.2. Determination of Anthocyanins

For a quantitative determination of anthocyanins, the method described by Oszmiański (2002) [49] was used. The previously prepared extracts or intestinal fraction or dialysate were filtered through a disposable syringe filter (0.45 µm). The HPLC system and column were the same as described in Section 3.4.1. Ten µL of the samples was eluted for 26 min at 25 °C with the following eluents: a 4.5% aqueous solution of formic acid (A) and an 80% solution of acetonitrile in a previous solution of formic acid (B) at a flow rate of 1.0 mL/min as follows: 0 min–0% A; 7 min–17% A; 15 min–17% A; 21 min–100% A; 26 min–0% A. The anthocyanins were quantified at 520 nm. The amount of anthocyanins was calculated as cyanidin 3-glucoside.

#### 3.4.3. Antioxidant Activity against ABTS^+•^ Radical

Determining the antioxidant activity against the ABTS^+•^ radicals was carried out according to the method described by Re et al. (1999) [50]. The solution of cationic radicals was prepared by combining 7 mM of ABTS^+•^ and 2.45 mM of potassium persulfate. The solution was left in a dark place for 18 h to form colored radicals. The solution was diluted with ethanol to obtain an absorbance measured at 734 nm with a UV/Visible Spectrophotometer (Ultrospec 2000, Pharmacia Biotech, Amersham, UK) between 0.740 and 0.750. One mg/mL solution of Trolox in ethanol was diluted into six 10-mL volumetric flasks, creating solutions in the following concentrations: 50, 100, 150, 200, 250, and 300 µg/mL to determine the calibration curves. Then, 0.025 mL of subsequent Trolox standards or samples were mixed with 2.5 mL of ABTS^+•^ radical solution and incubated at 30 °C for 6 min. The blank sample contained water instead of a sample extract. The antioxidant capacity was expressed as Trolox Equivalent Antioxidant Capacity (TEAC) per 1 mL of the sample.

#### 3.4.4. Antioxidative Activity against the DPPH^•^ Radical

The determination of the antioxidant activity against the DPPH^•^ radical was carried out according to the method presented by Yen and Chen (1995) [51]. A 1 mM stock solution of DPPH^•^ in 80% methanol was prepared and incubated in a dark place for 3 h to obtain a concentration of 0.1 mM and absorbance within 0.700–0.800 at 517 nm (Ultrospec 2000, Pharmacia Biotech). A standard curve was prepared from Trolox dissolved in methanol (1 mg/mL) in six 10 mL volumetric flasks, obtaining concentrations of 10, 20, 30, 40, 50, and 100 µg/mL, respectively. Then, 2 mL of radical solution and 0.1 mL of the test sample were mixed, and the absorbance was measured after incubation for 30 min in darkness. The antioxidant activity was expressed as Trolox Equivalent Antioxidant Capacity (TEAC) per 1 mL of the sample.

### 3.5. Statistical Analysis

The results obtained were subjected to a one-way analysis of the variance and an analysis of the statistical significance of the differences in mean values using the Tukey test at a confidence level of α = 0.05. Statistica 7.1 software (StatSoft, Tulsa, OK, USA) was used for data analysis. All technological procedures as well as digestion were carried out triplicate, whereas all analytical analyses were carried out in duplicate.

## 4. Conclusions

HPP as well as TP enabled the retention of vitamin C, anthocyanins, and high antioxidant capacity in blackcurrant products in almost all the samples. Digestion under salivary conditions caused a decrease in the vitamin C and anthocyanin concentration as well as the antioxidant capacity measured with DPPH^•^, which was connected with the changes in the natural pH of fruits in salivary digestion. On the other hand, both the vitamin C and anthocyanin concentration increased after stomach digestion, which can be justified by the low pH in the stomach. The first step in the oxidation of vitamin C and the transformation of anthocyanins into quinoidal bases and colorless hemiketals are reversible reactions and may be the reason why an increase of these compounds in the stomach was observed. As a result of further digestion in the small intestine and dialysate digestion stages, a very high degradation of each compound was noted, thus confirming other authors’ findings. The biological activity of vitamin C and anthocyanins may be related to the absorption of these compounds in the early stages of digestion or similar activity of their metabolites.

Despite the low stability of each compound during digestion, a higher bioaccessibility of vitamin C and anthocyanins after HPP treatment was observed. The higher the pressure used, the better bioaccessibility was observed, especially at the gastric stage. The lowest bioaccessibility of vitamin C and anthocyanins was observed in all the TP-treated samples at each of the digestion steps (except for the intestinal step for anthocyanins). The results of antioxidant capacity varied depending on the radical used in the assay (ABTS^+•^ and DPPH^•^). Antioxidant capacity against the ABTS^+•^ radical in the in vitro digestion model increased slightly after the simulation of salivary digestion; then, it reached the highest value at the stage of simulated gastric digestion, thereby indicating that the metabolites of the selected nutritional compounds from blackcurrants may indicate greater antioxidant potential.

A small number of papers on the impact of HPP on the bioaccessibility of hydrophilic antioxidants in food, as well as the promising results of this research, encourage their continuation. Further studies will be focused on determining selected metabolites in the nutritional compounds found in blackcurrants, as well as the use of more advanced methods to simulate the absorption of nutritional compounds and metabolites in more natural conditions, for example with Caco-2 cell lines.

## Figures and Tables

**Table 1:** Results of physicochemical analyses of blackcurrants at various stages of digestion.

	Sample Code	Non-Digested Sample	SD	Salivary Digestive Fraction	SD	Stomach Digestive Fraction	SD	Non-Dialysed Intestinal Fraction	SD	Dialysate Fraction	SD
L-Ascorbic Acid (AA) [mg/100 g]	M	229.48 ^a^	6.43	134.42 ^a^	3.89	205.42 ^a^	6.73	3.71 ^ab^	0.36	<1.0 ^b^	-
	P	227.24 ^a^	4.45	112.02 ^b^	5.08	177.62 ^c^	7.03	3.48 ^bc^	0.16	<1.0 ^b^	-
	TP	221.56 ^ab^	4.71	104.37 ^c^	5.80	133.66 ^d^	5.79	2.35 ^d^	0.37	<1.0 ^b^	-
	HPP200	229.20 ^a^	2.41	97.70 ^c^	4.26	140.95 ^d^	4.69	2.90 ^cd^	0.30	<1.0 ^b^	-
	HPP400	225.23 ^ab^	1.65	117.63 ^b^	2.67	189.17 ^bc^	5.36	4.08 ^ab^	0.33	2.20 ^a^	0.49
	HPP600	217.38 ^c^	6.92	117.02 ^b^	3.61	194.95 ^ab^	9.37	4.18 ^a^	0.50	2.09 ^a^	0.35
Sum of Anthocyanins [mg/kg]	M	736.70 ^a^	38.30	257.43 ^c^	12.99	454.69 ^a^	24.49	10.22 ^c^	1.25	11.86 ^a^	0.95
	P	638.04 ^b^	43.52	251.94 ^bc^	10.19	391.43 ^b^	21.53	3.07 ^d^	0.58	3.49 ^b^	0.91
	TP	601.98 ^b^	25.31	178.15 ^e^	9.56	330.07 ^c^	13.47	8.84 ^c^	0.75	1.53 ^c^	0.38
	HPP200	619.09 ^b^	42.13	220.23 ^d^	18.75	391.41 ^b^	15.87	14.10 ^b^	1.04	1.41 ^c^	0.47
	HPP400	626.05 ^b^	44.38	287.85 ^a^	13.91	408.71 ^b^	22.38	19.02 ^a^	1.33	1.77 ^c^	0.47
	HPP600	622.81 ^b^	43.22	277.29 ^ab^	16.04	419.47 ^ab^	24.03	4.64 ^d^	0.76	1.61 ^c^	0.40
ABTS^+•^ (µm/mL TEAC)	M	30.80 ^a^	0.81	32.48 ^b^	0.83	38.28 ^b^	1.95	12.81 ^a^	0.54	11.25 ^b^	0.42
	P	30.11 ^ab^	1.16	31.70 ^b^	0.78	35.86 ^c^	0.90	9.45 ^b^	0.06	9.31 ^c^	0.48
	TP	27.03 ^c^	1.49	28.38 ^c^	0.96	35.76 ^c^	1.18	2.94 ^e^	0.09	6.80 ^d^	0.12
	HPP200	27.95 ^c^	1.36	28.91 ^c^	1.17	35.30 ^c^	0.66	5.29 ^d^	0.23	9.34 ^c^	0.26
	HPP400	28.20 ^bc^	0.73	37.29 ^a^	1.03	42.02 ^a^	0.66	9.59 ^b^	0.39	13.44 ^a^	0.47
	HPP600	31.82 ^a^	1.59	34.91 ^a^	0.78	35.49 ^c^	0.66	6.25 ^c^	0.22	9.39 ^c^	0.37
DPPH^•^ (µm/mL TEAC)	M	45.72 ^a^	1.63	22.21 ^a^	1.17	25.81 ^b^	1.43	4.36 ^a^	0.20	4.41 ^a^	0.03
	P	47.24 ^a^	1.57	23.75 ^a^	0.96	26.08 ^ab^	0.55	4.22 ^ab^	0.14	3.33 ^cd^	0.08
	TP	45.11 ^a^	0.11	21.33 ^a^	0.82	21.99 ^c^	1.14	3.86 ^bc^	0.20	3.18 ^d^	0.05
	HPP200	47.35 ^a^	0.34	22.05 ^a^	0.54	22.67 ^c^	1.15	3.63 ^c^	0.15	3.45 ^c^	0.03
	HPP400	45.61 ^a^	0.28	22.63 ^a^	1.33	28.94 ^a^	0.94	4.63 ^a^	0.20	3.83 ^b^	0.01
	HPP600	45.89 ^a^	0.45	21.99 ^a^	1.00	27.15 ^ab^	1.36	4.56 ^a^	0.20	3.41 ^c^	0.11

M–fruit manually crushed in a mortar; P–unprocessed puree; TP–thermally pasteurized puree; HPP200–puree after HPP at 200 MPa; HPP400–puree after HPP at 400 MPa; HPP600–puree after HPP at 600 MPa; *n* = 3, small letters a–c, indicate statically significant differences in the mean values within a column at a confidence level of α = 0.05; SD–standard deviation. ABTS^+•^: 2,2′-azino-bis(3-ethylbenzothiazoline-6-sulfonic) acid diammonium salt, DPPH^•^: 2,2-diphenyl-1-picrylhydrazyl, TEAC: Trolox Equivalent Antioxidant Capacity.
